# Significant number of *Plasmodium vivax* mono-infections by PCR misidentified as mixed infections (*P. vivax/P. falciparum*) by microscopy and rapid diagnostic tests: malaria diagnostic challenges in Ethiopia

**DOI:** 10.1186/s12936-023-04635-x

**Published:** 2023-07-01

**Authors:** Abnet Abebe, Didier Menard, Sisay Dugassa, Ashenafi Assefa, Jonathan J. Juliano, Eugenia Lo, Lemu Golassa

**Affiliations:** 1 Aklilu Lemma Institute of Pathobiology, Addis Ababa University, Addis Ababa, Ethiopia; 2 Ethiopian Public Health Institute, Addis Ababa, Ethiopia; 3 Laboratory of Parasitology and Mycology, University of Strasbourg, Strasbourg, France; 4 Division of Infectious Disease, School of Medicine, University of North Carolina at Chapel Hill, Chapel Hill, North Carolina USA; 5 Department of Biological Sciences, Bioinformatics Research Center, University of North Carolina at Charlotte, Charlotte, North Carolina, USA

**Keywords:** Malaria, *Plasmodium vivax*, *P.vivax*/*P.falciparum* (mixed), Diagnostic method, Ethiopia

## Abstract

**Background:**

*Plasmodium vivax* malaria is now recognized as a cause of severe morbidity and mortality, resulting in a substantial negative effect on health especially in endemic countries. Accurate and prompt diagnosis and treatment of *P. vivax* malaria is vital for the control and elimination of the disease.

**Methods:**

A cross-sectional study was conducted from February 2021 to September 2022 at five malaria endemic sites in Ethiopia including Aribaminch, Shewarobit, Metehara, Gambella, and Dubti. A total of 365 samples that were diagnosed positive for *P. vivax* (mono and mixed infection) using RDT, site level microscopists and expert microscopists were selected for PCR. Statistical analyses were performed to calculate the proportions, agreement (k), frequencies, and ranges among different diagnostic methods. Fisher’s exact tests and correlation test were used to detect associations and relationship between different variables.

**Results:**

Of the 365 samples, 324 (88.8%), 37(10.1%), 2 (0.5%), and 2 (0.5%) were *P. vivax* (mono), *P. vivax*/*Plasmodium falciparum* (mixed), *P*. *falciparum* (mono) and negative by PCR, respectively. The overall agreement of rapid diagnostic test (RDT), site level microscopy and expert microscopists result with PCR was 90.41% (k: 0.49), 90.96% (k: 0.53), and 80.27% (k: 0.24). The overall prevalence of sexual (gametocyte) stage *P. vivax* in the study population was 215/361 (59.6%). The majority of these 215 samples (180; 83.7%) had below 1000 parasites/µl, with only four samples (1.9%) had ≥ 5000 parasites/µl. The gametocyte density was found to be weakly positive but statically significant with asexual parasitaemia (*r* = 0.31; *p* < 0.001).

**Conclusion:**

Both microscopy and RDT showed moderate agreement with PCR in the detection and identification of *P. vivax* (mono) and *P. vivax*/*P. falciparum* (mixed) infections. Therefore, to achieve malaria elimination goals, strengthening routine malaria diagnostic methods by implementing diagnostic tools with a good performance in detecting and accurately identifying malaria species in clinical settings is recommended.

## Background

Malaria is a life-threatening disease caused by parasites that are transmitted to humans through the bites of infected female *Anopheles* mosquitoes. According to World Health Organization (WHO) 2022 report, there were an estimated of 247 million cases and 619,000 deaths from malaria worldwide [1]. *Plasmodium vivax* is the most geographically widespread parasite and poses a serious threat to public health [2, 3]. In Ethiopia, about 75% of the land mass is malarious, putting around 60% of the population at risk [4]. The proportion of *Plasmodium falciparum* and *P. vivax* in Ethiopia was 62.8% and 37.2%, respectively [5]. In co-endemic areas, where intense malaria-control activities have reduced the burden of *P. falciparum*, there has been a rise in the proportion of malaria attributable to *P. vivax* [6]. *Plasmodium vivax* is a cause of malaria, associated with a significant public health burden [7, 8]. The dormant liver stages, that is, hypnozoites, may cause relapse infections over the course of weeks to years after clearance of the blood stage infection [9, 10]. The control and elimination of *P. vivax* is more challenging than that of *P. falciparum*, a reflection of key differences in parasite and vector biology [11]. Clear understanding of the distribution and clinical management of *P. vivax* is essential for informed decisions on appropriate control strategies to be designed and implemented against this neglected species.

Accurate and prompt diagnosis of malaria cases is key to the control and elimination of malaria [12–14]. The diagnosis of malaria using rapid diagnostic test (RDT) and microscopy have the advantages of being cheap, but these methods lack sufficient sensitivity to detect infections with low parasite density [15, 16], and often fails to identify a substantial fraction of *P. vivax* infections in blood [11]. In recent years, molecular diagnosis has emerged as the most sensitive and specific method for malaria diagnosis [17, 18]. Compared to real-time PCR assays, RDTs and microscopy are less effective for detecting low parasitaemia often seen in vivax infections [19]. Apart from the shortcoming in diagnostics, an increasing altitudinal range of mosquito distribution associated with climate change and human land use implies the potential for increased malaria burden across broader landscape, especially for countries of East Africa and South America [20].

Ethiopia has planned to eliminate malaria from specific geographic areas by 2020 and from the whole country by 2030 [21–23]. *Plasmodium vivax* distribution has been expanding to the highland, causing occasional malaria epidemics, although the existing deployed interventions seem to have an impact on the prevalence of this parasite [24].To achieve the elimination goal, the country must properly and consistently implement policies and practices which are used for the intervention of this neglected species. Poor diagnostic performance, low parasite density, the presence of gametocytes especially in asymptomatic infections, and the coexistence of different malarial species in the country hinder the progress towards elimination. So to achieve the elimination, sensitive diagnostic tools with good performance in detecting low parasitaemia infections and accurately identifying malarial species are necessary.

This study aims to evaluate the performance of routine laboratory diagnostic methods for the diagnosis of pure *P. vivax* infection and *P. vivax*/*P. falciparum* mixed infection (referred to as ‘mixed infection’ thereafter) in febrile samples collected from five malaria endemic sites in Ethiopia. These sites represent different transmission intensities and environmental characteristics such as altitude.

## Methods

### Study design and study participants

**Fig. 1:**
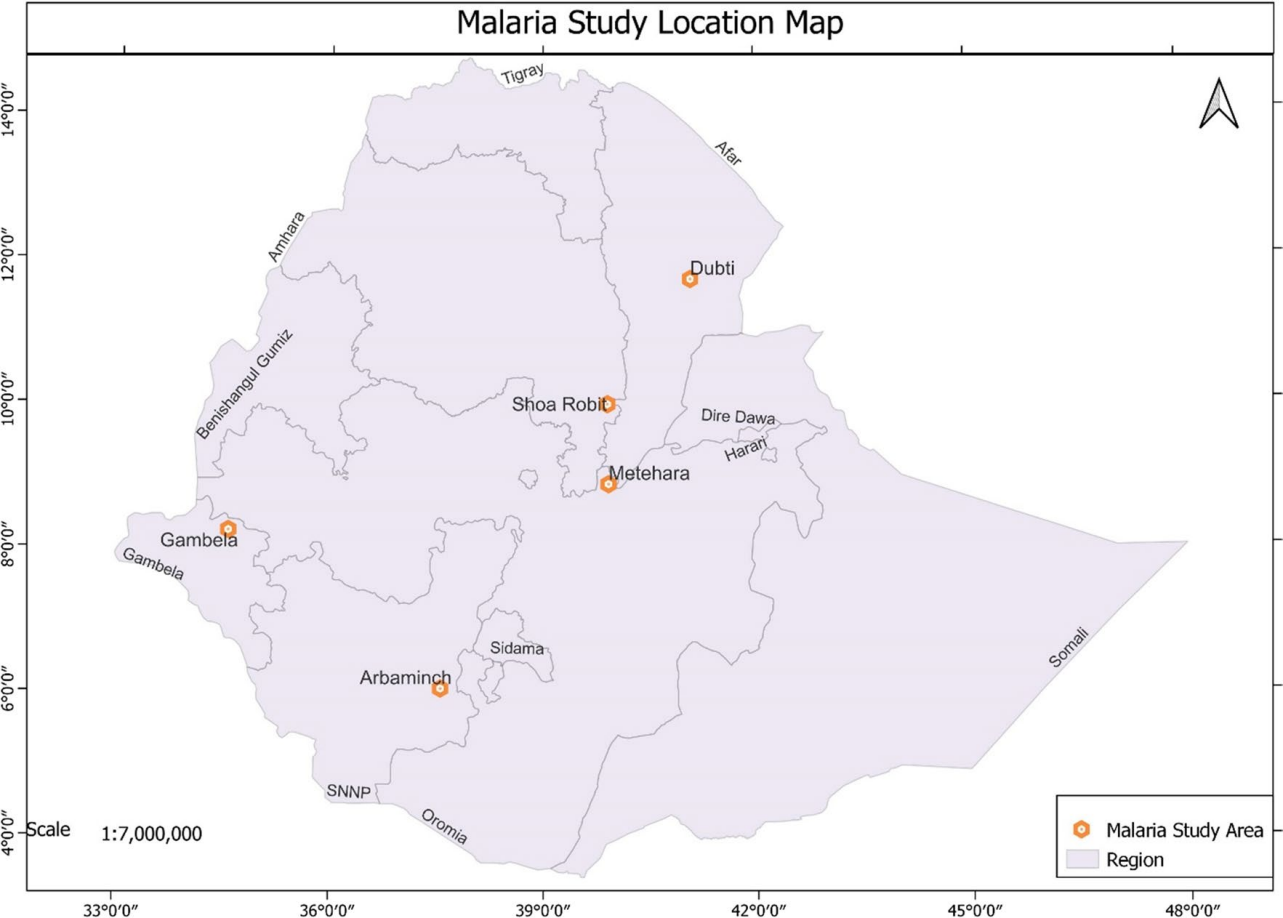
A map showing study sites, Ethiopia.

A cross-sectional study was conducted from February 2021 to September 2022 at five malaria endemic sites in Ethiopia. The study sites were selected from malaria endemic areas based on their *P. vivax* prevalence in the region and from different malaria risk stratification areas. The study sites include Aribaminch, Shewarobit, Metehara, Gambella, and Dubti **(**Fig. 1**)**. Arbaminch hospital is located in the Southern Nation and Nationalities People Regional State in the southern part of the country (altitude 1200 m); Dubti hospital is located in the Afar Regional State in the northern-east part of the country (altitude 379 m); Gambella hospital is located in Gambella Regional State in the western part of the country (altitude 447 m); Metehara hospital is located in Oromia Regional State in the eastern part of the country (altitude 959 m); and Shewarobit hospital is located in Amhara Regional State in the northern part of the country (altitude 1268 m).

### Sample size

Sample size determination was calculated using proportion of *P. vivax* (37.2%) from a total positive cases reported in Ethiopia(5). The following formula was used to calculate sample size:$n = z_{{\alpha \mathord{\left/ {\vphantom {\alpha 2}} \right. \kern-\nulldelimiterspace} 2}}^2 \times \frac{{p(1 - p)}}{{{\varepsilon ^2}}}$

P: proportion of *P. vivax*, Z: z- value at 5% level of significance, ε: margin of error, n: number of minimum sample size. Given that: p = 37.2%, Z = 1.96, and ε = 0.05. So the sample size calculated (n) was 358 and the total sample size with 10% non-response rate (N) was 394 (this is the sample collected at site level). The final sample size after re-examination of blood film slides at the central level was 365.

A total of 394 participants who were presented with malaria sign/symptom and diagnosed positive for pure *P. vivax* or mixed infection using both RDT (this method was used in the hospital only for this study) and microscopy were included in this study. Blood smears were re-examined blindly by expert microscopists at national level and samples which showed no *P. vivax* were excluded. A total of 365 samples that were diagnosed positive for *P. vivax* (mono or mixed infection) based on RDT, site level microscopists and expert microscopists were selected for PCR analysis.

### Study flow chart

**Fig. 2:**
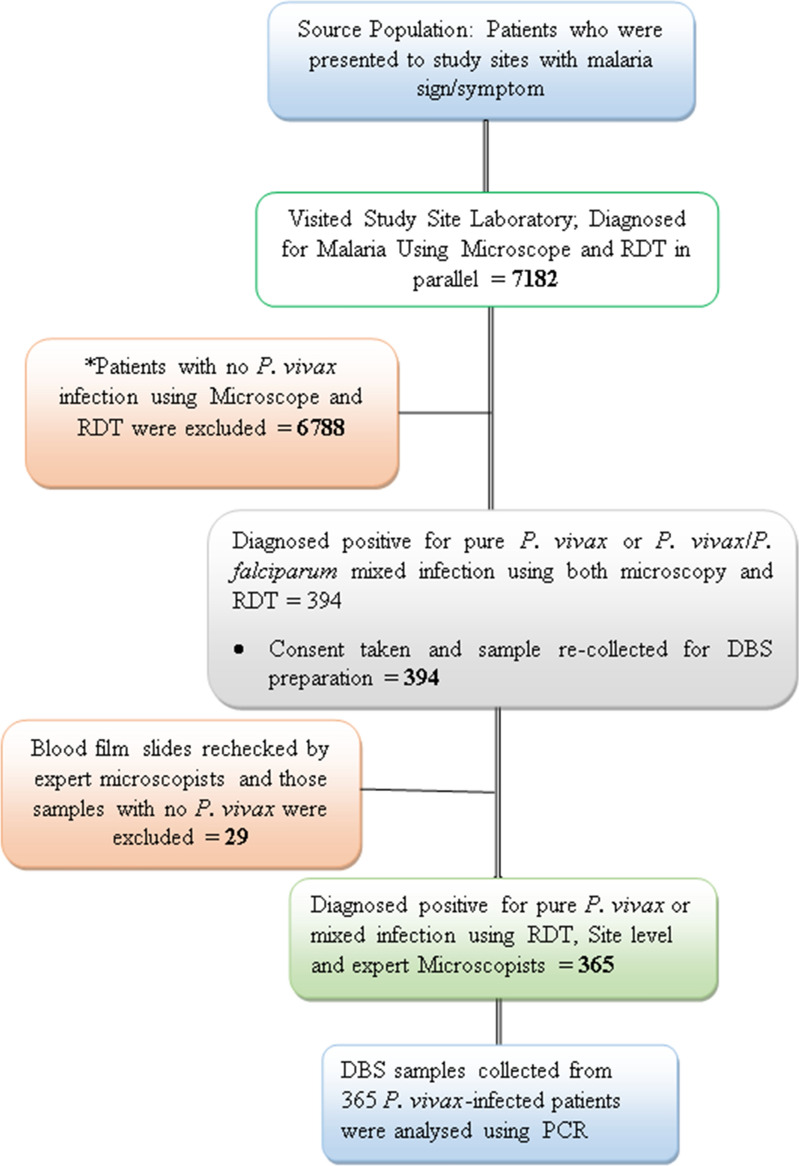
Flow chart of the study conducted from Feb. 2021 to Sep 2022 at five malaria endemic sites. *Patients with no P. vivax infection using Microscopy and RDT were excluded, because the aim was to assess the performance of methods on the diagnosis of pure P. vivax or P. vivax/P. falciparum (mixed) infection in Ethiopia

The flow chart of the study conducted from February 2021 to September 2022 at five malaria endemic sites in Ethiopia is indicated in Fig. 2.

### Laboratory diagnosis

#### Sample collection

Finger prick blood sample was collected from suspected malaria patients who were referred to the laboratory of study sites. A unique identifier was assigned for each study participant to trace them at any time. The sample collected from suspected malaria patients was used for RDT and blood smears preparation. Thick and thin blood smears were stained with 10% working Giemsa solution (pH 7.2) for 10 min. Blood film slides were examined with 100X objective using microscope. Malaria laboratory diagnosis was done using RDT and microscopy in parallel for each suspected malaria patients. Then those who were positive for pure *P. vivax* and mixed infection using both RDT and microscopy were enrolled in the study. Two millilitre (2 ml) of venous blood sample was collected from volunteer individuals based on the inclusion criteria. The collected sample was used for the preparation of thin and thick blood film (used by the facility for external quality assessment programme), and for the preparation of dried blood spots (DBS). The DBS samples were kept at − 20 °C till shipped to the University of Strasbourg, France, for molecular analysis.

#### Rapid diagnostic test

RDT kit currently implemented by the country is SD BIOLINE Malaria Ag *Pf/Pv* (05FK80). It is a rapid, qualitative test for the detection of HRP-II (Histidine-rich protein II) specific to *P. falciparum* and *Plasmodium* lactate dehydrogenase (pLDH) specific to *Plasmodium vivax*. Five microlitres (5 µl) of whole blood was used for the test and the result was interpreted at 15 min (up to 30 min).

#### Microscopy

Microscopic examination of Giemsa-stained thick and thin blood film was used for the detection, identification and quantification of malaria parasites. The first diagnosis using microscopy was done at study site by site level microscopists. Site level microscopists are microscopists who are providing routine laboratory diagnosis service at study site (employees of the study sites). Parasite counts was measured for both sexual and asexual stages on thick film and reported per 200 WBCs or parasite count per microlitre of blood, assuming a total white blood cell count of 8000/µl. The densities of asexual and gametocyte stages in peripheral blood were determined by WHO certified microscopists using microscopy at national level. The quantification of asexual stage parasite density was conducted for *P. vivax* on the blood film slides with pure *P. vivax* infection and the asexual stage of both species on the blood film slides with a mixed infection. Whereas the density of gametocyte was estimated only for the gametocyte of *P. vivax* on both blood film slides with pure *P. vivax* and mixed species. The parasite density of both the asexual and sexual stages were then classified as low (below 1000 parasites per microlitre of blood), intermediate (1000–4999 parasites per microlitre of blood) and high (≥ 5000 parasites per microlitre of blood) [25]. Blood film slides were declared no parasite seen after examination of at least 100 high-power microscope fields [26]. Blood film slides were rechecked and quantified by WHO-certified malaria microscopists (who are working as reference readers of blood film slides in the country).

#### DNA extraction

Genomic DNA was extracted from DBS collected from study participants using QIAamp DNA Extraction kit (Cat.No: 79,216, Lot: 172,018,338, Germany) based on manufacture instructions. Each genomic DNA was extracted from one punch (with a diameter of 6 mm) of DBS sample, and DNA was eluted using 100 µL of TE (Tris-EDTA) buffer, then the extracted DNA was kept at − 20 °C till PCR process.

#### Molecular diagnosis

Genomic DNA was extracted from DBS collected from study participants using QIAamp DNA Extraction kit (Cat.No: 79,216, Lot: 172,018,338, Germany) based on manufacture instructions. Each genomic DNA was extracted from one punch (with a diameter of 6 mm) of DBS sample, and DNA was eluted using 100 µL of TE (Tris-EDTA) buffer, then the extracted DNA was kept at − 20 °C till PCR process.

**Table 1:** Species-specific primers used for the detection and species identification of *P. vivax and P. falciparum*

Species	Forward or reverse	Primer
*P. vivax*	Forward	5′ —TGCTACAGGTGCATCTCTTGTATTC
	Reverse	5′ —ATTTGTCCCCAAGGTAAAACG
*P. falciparum*	Forward	5′ —ATGGATATCTGGATTGATTTTATTTATGA
	Reverse	5′—TCCTCCACATATCCAAATTACTGC

The PCR amplifications using Bio-Rad CFX96 Real-Time PCR detection system were carried out following 10 µL of Advanced mix sybgreen (2x) (Catalog # 1,725,271, Bio-Rad laboratories Inc. United State), 4 µL of molecular water, 0.5 µL of each primer (Table 1), and 5 µL of DNA template with the final volume of 20 µL.

#### Quality assurance

Training has been given for data and sample collectors. The clarity and flow of each question and the time to fill out the questionnaire were assessed. The collected data was checked for completeness by the principal investigator. The quality of kits and/or reagents which were used for all diagnostic methods have been assured using different quality control methods specific to each reagent and kit.

### Statistical analysis

All data were entered into Microsoft Office Excel and statistical analyses were performed with Statistical Package for Social Sciences (SPSS) version 25. Descriptive statistics were used to calculate proportions, agreement, frequencies, and ranges. Fisher’s exact tests and correlation test were used to detect associations and relationship between different variables, respectively. The strength of agreement between RDT, site microscopy, and expert microscopists with PCR were determined by Kappa (K) value. Kappa (K) value is classified as: 0.01–0.20 slight agreement, 0.21–0.40 fair agreement, 0.41–0.60 moderate agreement, 0.61–0.80 substantial agreement, and 0.81–1.00 almost perfect agreement [13, 29]. The strength of correlation was interpreted as; 0.00–0.10 Negligible correlation, 0.10–0.39 Weak correlation, 0.40–0.69 Moderate correlation, 0.70–0.89 Strong correlation, and 0.90–1.00 Very strong correlation [30]. The p-value < 0.05 was considered statically significant.

## Results

### Characteristics of the study participants

Females accounted for 35.1% (128/365) of the study participants. The mean age of the participants was 24.8 (ranging from 1 to 80 years old) with 137 (37.5%) of the participants age from 15 to 24 years old. Among all participants, 75 (20.5%) were farmer and 212 (58.1%) live in an urban area (Table 2).

**Table 2:** Socio demographic characteristics of study participants, Ethiopia (n = 365)

Variables	Category	Number	Percent %
Sex			
	Female	128	35.1
	Male	237	64.9
Age			
	< 5	15	4.1
	5–14	26	7.1
	15–24	137	37.5
	25–34	123	33.7
	≥ 35	64	17.5
Residence			
	Rural	153	41.9
	Urban	212	58.1
Marital status			
	Single	217	59.5
	Married	145	39.7
	Divorced	3	0.8
Study sites			
	Aribaminch	75	20.5
	Shewarobit	70	19.2
	Metehara	75	20.5
	Gambella	75	20.5
	Dubti	70	19.2

### Performance of diagnostic methods

For the 329 samples that were diagnosed as pure *P. vivax* by RDT, 310 (94.2%) were pure *P. vivax* infection and 17 (5.2%) were mixed infection based on PCR; whereas for the 36 samples diagnosed as mixed infection by RDT, 14 (38.9%) were pure *P. vivax* infection and 20 (55.6%) were *P. vivax*/*P. falciparum* mixed infection based on PCR.

For the 327 samples diagnosed as pure *P. vivax* infection by site level microscopists, 310 (94.8%) were pure *P. vivax* infection, 15 (4.6%) were mixed infection, 1 (0.3%) was pure *P*. *falciparum*, and 1 (0.3%) was negative using PCR. Among the 38 mixed infections diagnosed by site level microscopists, 14 (36.8%) were pure *P. vivax* infection, and 22 (57.9%) were mixed infection using PCR. Of the 68 samples diagnosed as mixed infections by expert microscopists, 49 (72%) were pure *P. vivax* infection, 18 (26.5%) were mixed infections and 1 (1.5%) was pure *P*. *falciparum* infection using PCR.

PCR was considered as a reference method for the evaluation of each the diagnostic methods. Of the 365 samples; 324 (88.8%), 37(10.1%), 2 (0.5%) and 2 (0.5%) were *P. vivax*, *P .vivax*/*P. falciparum*, *P*. *falciparum* and negative, respectively using PCR. The overall agreement of RDT, site level microscopy and expert microscopists result was 90.41% (K: 0495), 90.96% (k: 0.534), and 80.27% (k: 0.238), respectively (Table 3). The overall agreement of RDT and site level microscopy with expert microscopists result was 81.37% (K: 0249), and 82.19% (k: 0.286), respectively.

**Table 3:** Over all agreement of RDT and microscopy against real-time PCR as gold standard (n = 365)

Results		Reference Method (PCR)					Agreement	Kappa	Kappa Interpretation/agreement
		*Pv*	*Pv/Pf*	*Pf*	Negative	Total			
RDT									
*Pv*		310	17	1	1	329	90.41%	0.495	Moderate
*Pv/Pf*		14	20	1	1	36			
Total		324	37	2	2	365			
Site level Microscopists									
*Pv*		310	15	1	1	327	90.96%	0.534	Moderate
*Pv/Pf*		14	22	1	1	38			
Total		324	37	2	2	365			
Expert Microscopists									
*Pv*		275	19	1	2	297	80.27%	0.238	Fair
*Pv/Pf*		49	18	1	0	68			
Total		324	37	2	2	365			

Samples included in this study were those which were positive for *P.vivax* (Mono or mixed infection) using RDT and microscopy, so *P.falciparum* (mono) and negative results were not expected from RDT and Microscopy

For the 297 samples diagnosed as pure *P. vivax* infection by expert microscopists, 279 (93.9%) were pure *P. vivax* infection and 18 (6.1%) were mixed infection by RDT and site level microscopists, whereas from 68 samples diagnosed as mixed infection by expert microscopists, 50 (73.5%) and 48 (70.6%) samples were diagnosed as pure *P. vivax* infection by RDT and site level microscopists, respectively (Table 4).

**Table 4:** Over all agreement of RDT and site level microscopy against the result of Expert Microscopists (n = 365)

Results		Expert microscopists			Agreement	Kappa	Kappa interpretation
		*Pv*	*Pv/Pf*	Total			
RDT	*Pv*	279	50	329	81.37%	0.249	Fair
	*Pv/Pf*	18	18	36			
	Total	297	68	365			
Site level microscopists	*Pv*	279	48	327	82.19%	0.286	Fair
	*Pv/Pf*	18	20	38			
	Total	297	68	365			

Among all the five sites, Dubti (Afar) health facility had the lowest agreement in diagnostic results between the site level microscopists and PCR (81.43%), whereas the agreement of Shewarobit and Gambella hospitals had the highest agreement of 94.29% and 98.67%, respectively. In Dubti, among the 63 samples diagnosed as pure *P. vivax* infection by site level microscopists, 52 (82.5%), 10 (15.9%) and 1 (1.6%) were pure *P. vivax* infection, *P. vivax*/*P. falciparum* mixed infection and *P*. *falciparum* infection, respectively, based on PCR (Table 5). In Shewarobit, all the samples diagnosed as mixed infection by site level microscopists were *P. vivax* (mono) infection based on PCR.

**Table 5:** Agreement of site level microscopists result with reference method per study sites (n = 365)

			PCR Diagnosis and Identification					Agreement
	Study site name	Evaluated Results	*Pv*	*Pv/Pf*	*Pf*	Negative	Total	
Site level Microscopists Result	Aribaminch	*Pv*	70	0	0	0	70	93.33%
		*Pv/Pf*	4	0	0	1	5	
		Total	74	0	0	1	75	
	Shewarobit	*Pv*	66	0	0	0	66	94.29%
		*Pv/Pf*	4	0	0	0	4	
		Total	70	0	0	0	70	
	Metehara	*Pv*	74	1	0	0	75	98.67%
		*Pv/Pf*	0	0	0	0	0	
		Total	74	1	0	0	75	
	Gambella	*Pv*	48	4	0	1	53	86.67%
		*Pv/Pf*	5	17	0	0	22	
		Total	53	21	0	1	75	
	Dubti	*Pv*	52	10	1	0	63	81.43%
		*Pv/Pf*	1	5	1	0	7	
		Total	53	15	2	0	70	

Based on PCR, 324 of the samples were pure *P. vivax* infection and 37 were mixed infection. Of 324 pure *P. vivax* samples, 106 (32.7%) were reported from altitude below 500 m and 144 (44.4%) were from altitude of 1000–1500 m. For the 37 mixed infection samples, 36 (97.3%) were from altitude below 500 m and none were from altitude of 1000–1500 m. Pure *P. vivax* and mixed species identified using PCR were significantly associated with the altitude of study site (*p* < 0.001). Among the 37 mixed infection, 23 (62.2%) were from rural area, significantly higher than those 324 pure *P. vivax*, of which 129 (39.8%) were from rural area (*p* = 0.009; Table 6).

**Table 6:** Association of pure *P. vivax* and *P. vivax/P. falciparum* mixed infection with different variables (n = 361)

Variables		Pure *P. vivax* and *P. vivax/P. falciparum* mixed infection identified using PCR			P-Value
		*Pv*	*Pv/Pf*	Total	
Study site altitude	< 500 m	106 (32.7%)	36 (97.3%)	142	< 0.001
	500–1000 m	74 (22.8%)	1 (2.7%)	75	
	1000–1500 m	144 (44.4%)	0 (0%)	144	
	Total	324	37	361	
Age group (in years)	< 5	14 (4.3%)	1 (2.7%)	15	0.263
	5–14	22 (6.8%)	3 (8.1%)	25	
	15–24	117 (36.1)	18 (48.6%)	135	
	25–34	109 (33.6%)	13 (35.1%)	122	
	≥ 35	62 (19.1%)	2 (5.4%)	64	
	Total	324	37	361	
Residence area	Rural	129 (39.8%)	23 (62.2%)	152	0.009
	Urban	195 (60.2%)	14 (37.8%)	209	
	Total	324	37	361	
Sex	Female	113 (34.9%)	13 (35.1%)	126	0.975
	Male	211 (65.1%)	24 (64.9%)	235	
	Total	324	37	361	

### Asexual and sexual parasite density

From a total of 365 samples identified as pure *P. vivax* and mixed infections by RDT, 329 were pure *P. vivax* and 36 were mixed infections. Of 329 pure *P. vivax*, the asexual parasite density of 53 (16.1%), 124 (37.7%), and 152 (46.2%) samples had the parasite density below 1000 parasites/µL, between 1000 and 4999 parasites/µL, and ≥ 5000 parasites/µL, respectively. Whereas from 36 samples with *P. vivax*/*P. falciparum* mixed infections, the asexual parasite density of 9 (25%), 8 (22.2%), and 19 (52.8%) samples had the parasite density below 1000 parasites/µL, between 1000 and 4999 parasites/µL, and ≥ 5000 parasites/µL, respectively. The asexual stage parasite density has no significant association with the RDT Result (*p* = 0.138).

For the 361 samples identified as pure *P. vivax* and mixed infections by PCR, the mean asexual (the density of both species was quantified in a mixed infection) parasite density was 11,296 parasites/µL (ranging from 44 to 68,880 parasites/µL). The asexual parasite density of 60 (16.6%) samples were below 1000 parasites/µL, 131 (36.3%) were between 1000 and 4999 parasites/µL, and 170 (47.1%) were ≥ 5000 parasites/µL. For the 60 samples with an asexual stage parasite density below 1000 parasites/µL, 131 samples with an asexual stage parasite density between 1000 and 4999 parasites/µL, and 170 samples with an asexual stage parasite density ≥ 5000 parasites/µL, 4 (6.7%), 11 (8.4%) and 22 (12.9%) were mixed infection, respectively.

**Fig. 3:**
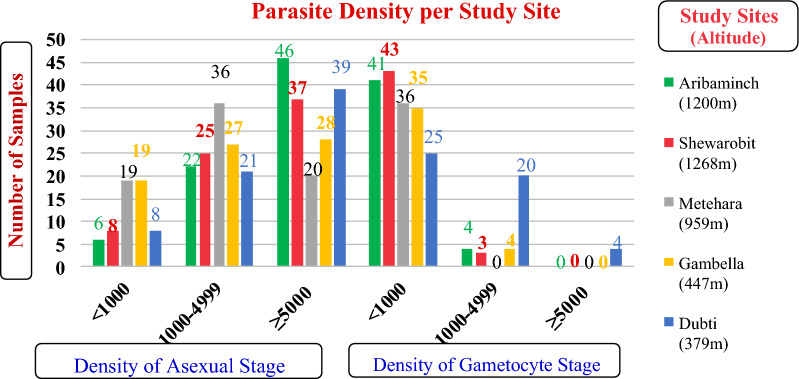
Asexual and gametocyte densities by study site.

**Fig. 4:**
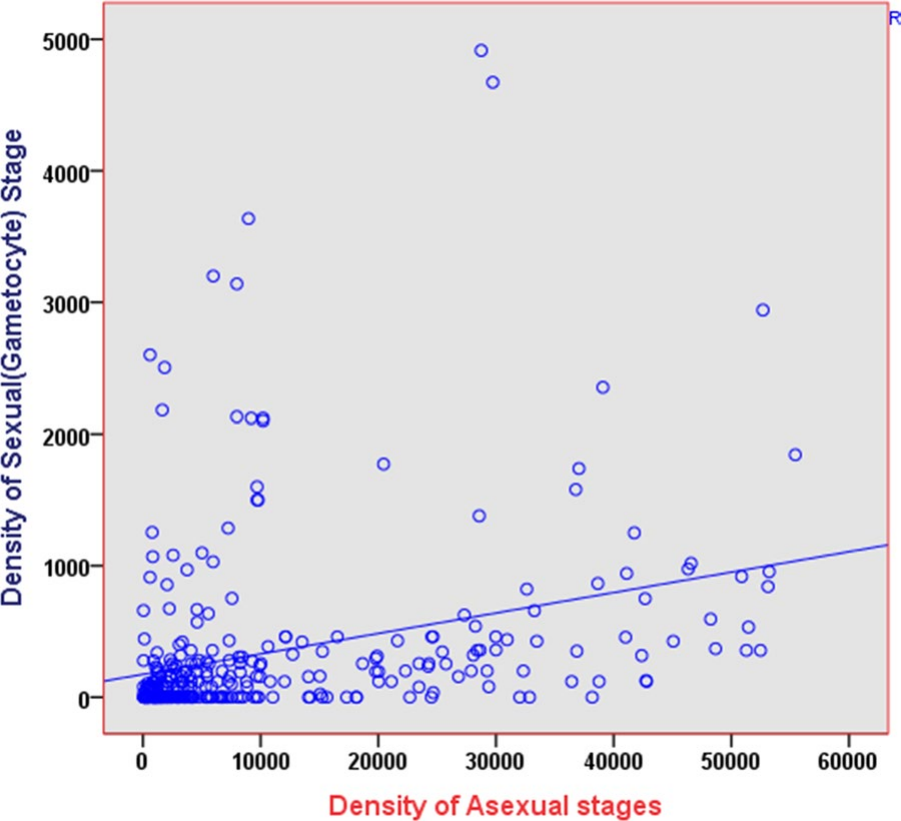
Correlation between asexual parasitemia and gametocytemia in pure *P. vivax* infections

The overall prevalence of sexual (gametocyte) stages of *P. vivax* was 215/361 (59.6%). Among these 215 samples with gametocyte, 180 (83.7%) were below 1000 parasites/µL, 31 (14.4%) were between 1000 and 4999 parasites/µL, and 4 (1.9%) were ≥ 5000 parasites/µL. For the 180 samples with a sexual stage parasite density below 1000 parasites/µL and from 31 samples with a sexual stage parasite density between 1000 and 4999 parasites/µL, 16 (8.9%) and 5 (16.1%) were mixed infection, respectively. For the 60 samples with an asexual stage parasite density below 1000 parasites/µL, 6 (10.0%) were from Aribaminch and 19 (31.7%) were from Gambella. For the 131 samples with an asexual stage parasite density between 1000 and 4999 parasites/µL, 36 (27.5%) were from Metehara, which was higher than Dubti 21 (16.0%). For the 180 samples with a sexual stage parasite density less than 1000 parasites/µL, 23.9% were from Shewarobit, which was higher than Gambella (19.4%) and Dubti (13.9%). Both an asexual and sexual stage parasite density were significantly associated with the study site (*p* < 0.001) **(**Fig. 3). To avoid any bias in parasite density due to mixed infection, only 324 samples that were diagnosed as pure *P. vivax* infection by PCR were used to test for the correlation between asexual and sexual stage densities. The mean density of asexual parasite was 10,496 parasites/µL (95% confidence interval: 9106−12,020 parasites/µL), and that of sexual (gametocyte) parasite was 337 parasites/µL (95% confidence interval: 266–417 parasites/µL). A weak correlation (r) was detected between asexual and gametocyte stages density among the samples (r = 0.314 with 95% confidence interval: 0.214–0.422, p< 0.001) (Fig. 4)  

## Discussion

Accurate and prompt laboratory diagnosis of malaria cases caused by *P. vivax* and other *Plasmodium* species is key to the control and elimination of this disease [31]. Previous studies have demonstrated higher sensitivity and specificity of real time-PCR based techniques compared to microscopy and RDT for the diagnosis of malaria parasite [32].

In this study, the overall agreement of RDT with PCR was 90.41%, which was higher than a study conducted in eastern Sudan with the agreement 81.2%(33), and Zambia 84.6% [33], but lower than 96.67% reported in China-Myanmar [34] and 97.6% in United Republic of Tanzania [35]. The inter assay agreement determined by Cohen’s Kappa coefficient of RDT and PCR in this study was k = 0.495, denoting (moderate agreement.), this was a similar level of agreement strength with some studies including work in Ghana with k = 0.47(moderate agreement) [36], but much lower than other studies conducted in Iran (k = 0.695; substantial agreement) [37], Cameroon (k = 0.71; substantial agreement) [38], Iran (k = 0.79; substantial agreement) [39], and Bangladesh (k = 0.80; substantial agreement) [19]. These findings showed that the agreement between RDT and PCR was not perfect likely due to low sensitivity and specificity of RDTs in detection and identification of *Plasmodium* species, suboptimal storage condition that affects the diagnostic quality of the kits, and/or lack of competency by laboratory personnel in the result interpretation.

Compared to the site level microscopists, the expert microscopists who were certified by WHO showed a lower agreement with PCR. The agreement of site level microscopy with PCR was 90.96%, which was relatively higher than a study conducted in Addis Ababa, Ethiopia with the percent agreement of 71.4% [40], Adama, Ethiopia 77.3% [32], but relatively lower than the study findings reported from; Hawassa Town, Southern Ethiopia 88% [41], rechecking laboratories in Ethiopia 96.8% [13], Zambia 91.3% [33], United Republic of Tanzania 93.5% [35], and eastern Sudan 96.1% [42]. The strength of agreement between these two diagnostic methods was moderate, slightly higher than that reported in Ghana (k = 0.40) [36] and almost similar with that in Cameroon (k = 0.54) [38], but relatively low compare to that in Iran (k = 0.714) [37, 39] and Bangladesh (k = 0.84) [19]. Even though microscopy is an easy, cheap, simple, and the gold standard method for malaria diagnosis [37], the agreement of this method with PCR was moderate. This may be due to low parasitaemia of samples, errors by microscopists, as well as poor quality of reagents and equipment. Although microscopy is the gold standard, missing mixed infections may lead to treatments that would fail to clear the presenting blood-stage infection [43]. Poor performance on detection and/or species identification, such as reporting *P. vivax* /*P. falciparum* mixed infection as a mono-infection can lead to inappropriate treatment.

Our findings showed that *P. vivax*/*P. falciparum* mixed infection is higher at low altitude, and that most of the identified *P. falciparum* infected samples were from sites at low altitude, similar to a prior study conducted in Northeastern Tanzania confirming that *P. falciparum* prevalence had a negative relationship with altitude [44]. The increasing evidence on the transmission of *P. vivax* in the areas traditionally considered as malaria free is an indication of the expansion of malaria transmission in Ethiopia to higher altitude settings [24], this may be due to local environmental modifications or expansion of mosquito’s habitat to non-endemic regions; besides changing human settlement pattern.

In this study, the correlation between asexual and gametocyte stage density was significant but weakly positive correlation, given that a previous study conducted in Brazil showed a moderate correlation across the range of parasite densities observed in *P. vivax*-infected blood donors [45], whereas the studies conducted in Thailand [46], Indonesia [47], and western Thailand and northern Peru [48] reported a significant and strong correlation between gametocyte density and asexual parasitemia. This difference may be explained by the competency of microscopists or the performance of the method used to properly differentiate and quantify different stages of the parasite.

This study was largely limited by its study population. The study population were only malaria patients who were diagnosed with pure *P. vivax* and mixed infections. It lacks negative samples, which is important to evaluate diagnostic method performance on the identification of true negative. So this made us challenging to evaluate the performance of the methods using different parameters like sensitivity and specificity. The other limitation of this study was conducting parasite density estimation only by microscopists using microscope. It may be a challenge to getting the exact parasite density because of the competency of microscopists.

## Conclusion

Both microscopy and RDT showed moderate agreement with PCR in the detection and identification of pure *P. vivax* and *P. vivax*/*P. falciparum* mixed infections. Even though microscopy and RDT are used routinely for malaria laboratory diagnosis in Ethiopia, a substantial number of *P. vivax* mono-infections are misidentified as mixed infections and such an outcome could affect the anti-malarial drug treatment regime. Therefore, to achieve malaria elimination goals, it is recommended to strengthen routine malaria laboratory diagnostic methods by implementing diagnostic tools with a good performance in detecting and accurately identifying malaria species in clinical settings, providing adequate in-service technical training and strengthening the external quality assessment programme.

## Data Availability

All relevant data are within the manuscript. The data that support the findings of this study are available from the corresponding author on reasonable request.

## Abbreviations

ALIPB: Aklilu lema Institute of pathobiology
DBS: Dried blood spots
DNA: Deoxyribonucleic acid
EPHI: Ethiopian Public Health Institute
HRP: Histidine-rich protein
IRB: Institutional review board
MoE: Ministry of education
NSP: 5National strategic plan
pLDH: *Plasmodium* *lactate* dehydrogenase
Pv/Pf: *Plasmodium vivax* and *Plasmodium falciparum*
RDT: Rapid diagnostic tests
RT PCR: Real time polymerase chain reaction
SOP: Standard operating procedures
SPSS: Statistical package for social sciences
WBC: White blood cells
WHO: World Health Organization
